# Factors associated with the successful completion of a substance rehabilitation programme at a psychiatric training hospital

**DOI:** 10.4102/sajpsychiatry.v26i0.1255

**Published:** 2020-02-03

**Authors:** Justine Dreyer, Jacobeth M. Pooe, Loveness Dzikiti, Christa Krüger

**Affiliations:** 1 Department of Psychiatry, Faculty of Health Sciences, University of Pretoria, Pretoria, South Africa; 2 School of Health Systems and Public Health, Faculty of Health Sciences, University of Pretoria, Pretoria, South Africa

**Keywords:** substance rehabilitation, psychiatric inpatients, dual diagnosis, co-occurring disorders, substance use disorders, mental illness, addiction treatment

## Abstract

**Background:**

Comorbid psychiatric and substance use disorders are common and present several treatment challenges.

**Aim:**

The aim of this study was to determine which patient and substance factors are associated with the completion of a substance rehabilitation programme in psychiatric inpatients.

**Setting:**

The study was conducted at the Substance Rehabilitation Unit (SRU) at Weskoppies Hospital, a psychiatric training hospital in South Africa, which offers a 6-week programme at the hospital for psychiatric inpatients.

**Methods:**

This descriptive, retrospective hospital-based study was carried out comparing completers and non-completers of the SRU programme with respect to patient and substance factors. All patients accepted into the SRU during 2013–2014 were included (*n* = 119). Data were collected over a year (2016–2017) from the clinical files, SRU referral forms, SRU attendance register, hospital computerised demographic records, nursing notes and administration files using a data collection sheet designed by the researchers for this study. Comparison between completers and non-completers was performed using Chi-Square or Fisher’s Exact tests.

**Results:**

The SRU accepted 119 patients from January 2013 to December 2014. The majority of the sample were involuntary patients (*n* = 39), 30–49 years old (*n* = 57), male (*n* = 89), unmarried (*n* = 112), never having received a disability grant (*n* = 27), unemployed (*n* = 96) and with a Grade 8–11 education (*n* = 49). Substance-induced psychotic disorders (*n* = 39), schizophrenia (*n* = 29) and bipolar disorders (*n* = 22) were found to be common. Frequent medical comorbidities included head injury (*n* = 27), cardiovascular disease (*n* = 18) and HIV reactivity (*n* = 7). Cannabis (*n* = 98), alcohol (*n* = 94) and nicotine (*n* = 90) were the most frequently used substances. Level of education (*p* = 0.004), disability grant status (*p* = 0.004), Nyaope use (*p* = 0.001) and nicotine use (*p* = 0.049) were statistically seen to be significantly associated with completion. Psychiatric diagnoses and general medical comorbidity were not associated with completion.

**Conclusions:**

This study has yielded several results in areas that have not yet been well researched in South Africa. Risk factors for non-completion may include lower levels of education, being on a disability grant and using Nyaope or nicotine, but may vary in different settings. Future research should focus on identifying further factors that may affect completion of substance rehabilitation in psychiatric inpatients, the role of disability grants in patients with co-occurring disorders and the effect of Nyaope and nicotine use on treatment outcomes in this population. Effective and accessible interventions to assist vulnerable patients also need to be identified.

## Introduction

The relationship between substance use and mental illness is complex. The two conditions can in some cases be unrelated, but more often they share similar risk factors or have a bidirectional interaction with each other.^1,2,3,4^ Fifty-one per cent of psychiatric inpatients in South Africa have a comorbid substance use disorder (SUD),^5^ whilst 57% of private substance rehabilitation centre clients have at least one comorbid psychiatric disorder.^2^ International rates of comorbidity are highly variable, in large part because of methodological differences, and range from 20% to 93% in substance rehabilitation centres.^6^ Patients with co-occurring substance use and psychiatric disorders are referred to as having ‘dual diagnosis’ or ‘co-occurring disorders’ (COD).^2,7^ They have a poorer prognosis than those with either disorder on its own, with greater interpersonal conflicts and aggression, more legal complications, higher suicide rates, and greater unemployment and financial problems.^2,5^

For the purposes of this study, we defined substance rehabilitation as any attempted completion of a structured medical, psychoeducational or psychotherapeutic treatment programme aimed at achieving abstinence and recovery from substance misuse and addiction. As such, it may include inpatient and outpatient detoxification and rehabilitation services as well as the medically guided use of agonist or aversive medications.

Substance rehabilitation in patients with COD is more complex, recovery is often prolonged and treatment is complicated by poor insight, poor compliance, poor engagement and higher relapse rates, often associated with greater treatment costs and caregiver burden.^1,2,4,5,7,8,9,10,11^ In spite of the complex treatment needs and challenges of COD patients, and perhaps because of them, effective treatment in South Africa and internationally is limited. Patients with COD usually pose treatment difficulties, especially when both the SUD and mental disorder are severe, and sequential treatment services often do not adequately meet the needs of COD patients, with some patients not being able to receive treatment at all.^2,5,10,12,13^ Resource constraints, lack of training for health care workers and lack of knowledge about the complexities associated with COD contribute to the lack of specialised treatment services.^13^ Integrated, comprehensive and individualised substance rehabilitation treatment is therefore needed for many COD patients, whereby each patient’s combination of substance use patterns, psychiatric diagnosis, level of motivation and level of functioning is considered.^2,5,7,12,13,14^ Such treatment allows vulnerable patients to receive the support they need to increase their chances of treatment completion and of receiving the full benefit of treatment.

An essential component of individualised care is to understand and anticipate the factors that might affect treatment completion. This knowledge can guide service planning and improve effectiveness of existing and future substance rehabilitation programmes for psychiatric inpatients by exposing treatment gaps and the areas where effort and resources should be focussed. It can improve the process of service adaptation to meet the needs of COD patients in a better way. It can also help to curb the inefficient use of limited treatment resources and direct future research.^15^ Although treatment completion does not necessarily equal treatment success, it has been found to consistently lead to more favourable outcomes such as abstinence and fewer relapses, higher rates of employment and lower rates of crime. Treatment non-completion, on the other hand, is associated with a higher risk of relapse, readmissions, legal problems and health problems.^16^ Internationally, substance rehabilitation drop-out rates are highly variable, ranging from 0% to 80%, and differ depending on setting, country, study population, study methodology and study duration.^16^ It does not appear that COD patients have higher rates of treatment non-completion than the general population.^6^ It is unknown whether COD patients in South Africa have higher rates of treatment non-completion than the 2% – 40% drop-out rate in general substance rehabilitation centres locally.^17^

Treatment completion is affected by the complex interplay between service factors, patient factors and substance factors.^16,18^ These associations should not be assumed to be causal, as there may be shared reasons for both non-completion and poor outcome.^16^ Younger age, cognitive dysfunction, personality disorder and low therapeutic alliance are amongst the few factors consistently found to predict drop-out in general substance rehabilitation.^6,16^ A recent systematic review reported mixed findings regarding the association of specific substances with treatment completion.^16^ South African studies suggest poor outcomes for many substances, in particular Nyaope^19^ and crystal methamphetamine (TIK).^11,20^ Nyaope is a very addictive and easily accessible South African street drug, which is highly associated with poverty, unemployment and incomplete schooling.^7,17,19,21,22,23^ It is composed of opiates, amphetamines and caffeine, with possible additional substances such as paracetamol, antiretrovirals, sedatives, anaesthetics, dextromethorphan, antibiotics and bulking agents (such as sand, soil and cement). It is usually smoked together with cannabis.^21,23,24^ The effects of the combination of substances contained in Nyaope have not been studied extensively and may interact with each other in both synergistic and opposing manners.^21,22^ Rehabilitation is reported to have little effect on Nyaope use, with many users relapsing soon after.^19^

With regard to factors affecting treatment completion in the COD population specifically, the literature is limited.^16^ The aim of this study was therefore to determine the completion rate and socio-demographic factors, psychiatric diagnoses, general medical conditions and patterns of drug use that are associated with completion of a substance rehabilitation treatment programme in psychiatric inpatients.

## Methods

### Study design

The study was a descriptive, retrospective, hospital-based two-group, cross-sectional study. Completers and non-completers of a Substance Rehabilitation Unit (SRU) programme for patients with co-occurring disorders (COD) were compared with respect to various socio-demographic factors, psychiatric diagnoses, medical comorbidities and substance use patterns.

### Setting

The study was conducted at the SRU at Weskoppies Hospital, which offers substance rehabilitation for inpatients at the hospital with COD. The SRU was established in 2011 after it became necessary for revolving door patients whose frequent relapses were linked to substance abuse and who often did not access substance rehabilitation services after discharge (sequential treatment). The programme follows a parallel treatment model for COD, that is, substance rehabilitation treatment and psychiatric management are performed by different treating multidisciplinary teams (MDTs) at the same time. Patients remain resident in the wards that they were in at the time of referral to the SRU, and attend the day programme each weekday at the SRU. Management of psychiatric conditions and overall case management remains the responsibility of the referring MDT. The programme entails daily group and individual sessions centred on substance education and group therapy, where patients are given support and the tools that they need for recovery and relapse prevention. Families are involved through a family support group. The programme is run by an MDT consisting of a psychiatrist, social worker, psychologist, occupational therapist, physiotherapist, dietician, nursing staff, pastoral care and external sponsors such as Alcoholics Anonymous. The programme runs on a 6-week cycle with five to eight patients at a time. Aftercare is provided in the form of outpatient support groups twice a month. The criteria for inclusion into the SRU are inpatient status, being between the ages of 18–65, able to read and write, understand the English language, apsychotic, non-violent and preferably in an open ward. Patients with cognitive impairment, aggression, mania and overt psychosis are excluded from the SRU. Only patients who are willing to attend the SRU and apparently motivated to abstain from substances are accepted. Voluntariness for substance rehabilitation is considered separately from willingness to accept psychiatric treatment at the time of admission when MHCA status is usually determined (see outcome measures, demographic variables). The legal status as per the definitions in the *Mental Health Care Act No. 17 of 2002* of South Africa (MHCA) is therefore not considered for acceptance into the SRU, so that this does not delay or become a barrier to accessing treatment. Treatment, therefore, remains the responsibility of the referring team. Patients may be expelled from the programme for disobeying SRU rules, including substance use and dealing, wilful damage to property, possession or weapons, violent or aggressive behaviour, or engaging in sexual or romantic relationships during sessions. Using the SRU inclusion and exclusion criteria as a guide, potential candidates for the SRU are referred to the SRU by their treating MDT by means of a detailed referral form. They are then assessed by the SRU MDT for suitability and readiness, before being accepted into the SRU. Non-completers of the SRU are managed further by the referring MDT in an individualised case-by-case manner, depending on the status of their psychiatric conditions.

### Study population and sampling strategy

All the patients who were accepted into the SRU during the 2-year period from January 2013 to December 2014 were included in this study. There were no other inclusion or exclusion criteria; a convenience sampling method was therefore utilised. The SRU was started in 2011 and needed time to stabilise and overcome the initial start-up challenges before effectiveness could be reliably assessed.

### Data collection

Data were collected from the clinical files, SRU referral forms, SRU attendance register, hospital computerised demographic records, nursing notes and administration files. All data were recorded on data sheets designed by the researchers for use in this study. All data were collected retrospectively by one researcher. Data collection was carried out over a 1-year period from 2016 to 2017.

### Outcome measures

#### Completion status

The primary outcome measure was completion status, which refers to whether or not a patient completed the full 6 weeks duration of the SRU programme. In a few cases, the patient disobeyed rules or relapsed and was temporarily suspended. The patient was able to complete after rejoining the programme in the next cycle.

#### Demographic variables

Demographic variables included age, gender, highest level of education, marital status, employment status and legal status under the MHCA.^25^

*Age* refers to the age of the individual at the time of referral to the SRU. *Highest level of education* refers to the highest completed level of education, categorised in the same way as the South African Stress and Health Survey (SASH).^26,27^ A best approximation was applied in cases where a patient attended a non-South African schooling system. *Marital status* was recorded as per clinical records and categorised as married, single, widowed, divorced, separated and other. *MHCA status* refers to the legal status (at the time of admission) of an individual receiving care, treatment or rehabilitation under the MHCA, and can include voluntary, assisted, involuntary and state patients. Voluntary, assisted and involuntary mental health care users (MHCUs) are patients who have a mental illness and require treatment. Voluntary MHCUs are able to make an informed decision and are willing to receive care, treatment and rehabilitation. Assisted and involuntary MHCUs are unable by way of mental illness to make an informed decision for care, treatment and rehabilitation related to mental illness, and are differentiated according to whether they do not oppose such treatment (assisted) or refuse and are a risk to themselves, others or their reputation (involuntary). State patients are criminal offenders who have been charged with a crime, found to be unfit to stand trial and/or not criminally responsible because of a mental illness or disability, and who have been referred by the court to a psychiatric facility for further inpatient treatment until reclassified or discharged by the judge in chambers.^25,28^

#### Medical comorbidities

Data on all recorded current general medical diagnoses were collected, including HIV status and pregnancy, amongst others.

#### Psychiatric diagnoses

Psychiatric diagnoses reflect the working Diagnostic and Statistical Manual of Mental Disorders, fourth edition, text-revised (DSM-IV-TR)^29^ diagnoses as recorded in the clinical files, and included primary psychiatric disorders, substance-induced disorders and personality disorders. Where the notes made specific mention of personality ‘traits’ but did not commit to the diagnosis of a personality disorder, this was recorded separately.

#### Previous substance rehabilitation

Data on previous substance rehabilitation attempts were recorded as per clinical files.

#### Pattern of substance use

Variables included the type of substance, number of substances and age of debut.

#### Reasons for non-completion

Reasons for non-completion were captured for patients who did not complete the SRU programme. More than one reason may have been applicable.

### Data analysis

All data were categorical, including age, which was divided into age categories similar to firm allocation at Weskoppies Hospital to aid data analysis. Frequency tables were compiled for each variable. Comparison between completers and non-completers was performed using Chi-Square or Fisher’s Exact tests. When missing data were encountered, cases were excluded for analyses of that variable only and sample sizes were adjusted for that variable. To avoid reducing the total sample size, cases were still included in the analysis for other variables. Statistical analysis was carried out using Stata 15.

### Ethical considerations

Permission to access patient records was obtained from the CEO of Weskoppies Hospital. Ethics approval and a waiver of informed consent were obtained from the University of Pretoria’s Faculty of Health Sciences Research Ethics Committee prior to commencing data collection (Ethical Clearance number: 30/2016).

## Results

### Descriptive statistics

During the 2-year period, 119 patients were accepted into the SRU, of which 76% (*n* = 90) completed and 24% (*n* = 29) did not complete the 6-week programme. The most common reason for non-completion was disobeying unit rules (*n* = 19), which included substance use during admission (*n* = 9). This was followed by lack of continued motivation (*n* = 7) and abscondment (*n* = 4).

Table 1 shows the demographic characteristics of the patients accepted into the SRU. Psychiatric and medical diagnoses are shown in Table 2 and substance use frequencies are shown in Table 3.

**TABLE 1:** Demographic characteristics of all patients accepted into the Substance Rehabilitation Unit during 2013–2014 (***n*** = 119).

Demographic characteristic	Variable	Accepted (*n* = 119)†		Completers (*n* = 90)†		Non-completers (*n* = 29)†	
		*n*	%	*n*	%	*n*	%
MHCA status	Voluntary	16	14.5	13	15.5	3	11.5
	Assisted	34	30.9	24	28.6	10	38.5
	Involuntary	39	35.5	27	32.1	12	46.2
	State patients	21	19.1	20	23.8	1	3.9
Age (years)	18–29 years	51	42.9	33	36.7	18	62.1
	30–49 years	57	47.9	48	53.3	9	31.0
	> 50 years	11	9.2	9	10.0	2	6.9
Gender	Male	89	74.8	64	71.1	25	86.2
	Female	30	25.2	26	28.9	4	13.8
Marital status	Married	3	2.5	1	1.1	2	6.9
	Unmarried	112	94.1	86	95.6	26	89.7
	Single	86	72.3	66	73.3	20	69.0
	Widowed	2	1.7	2	2.2	0	0
	Divorced	18	15.1	14	15.6	4	13.8
	Separated	6	5.0	4	4.4	2	6.9
	Other	4	3.4	3	3.3	1	3.5
Disability grant	Never received	27	71.1	25	83.3	2	25.0
	Currently receiving	10	26.3	5	16.7	5	62.5
	Previously received	1	2.6	0	0	1	12.5
Employment status	Employed	13	10.9	8	8.9	5	17.2
	Unemployed	96	80.7	73	81.1	23	79.3
	Student	6	5.0	6	6.7	0	0
	Informal work	3	2.5	2	2.2	1	3.5
	Retired	1	0.8	1	1.1	0	0
HLOE†	Grades 1–7	6	5.0	1	1.1	5	17.2
	Grades 8–11	49	41.2	35	98.9	14	48.3
	Matric	46	38.7	38	42.2	8	27.6
	Tertiary qualification	18	15.1	16	17.8	2	6.9

MHCA, *Mental Health Care Act No. 17 of 2002 of South Africa (2002)*^13^; HLOE, highest level of education.

† , Sample size was adjusted (and is therefore smaller) for variables with missing data. Percentages have been calculated according to adjusted sample sizes.

**TABLE 2:** Psychiatric and medical diagnoses of all patients accepted into the Substance Rehabilitation Unit during 2013–2014 (*n* = 119).

Diagnoses	Accepted (*n* = 119)		Completers (*n* = 90)		Non-completers (*n* = 29)	
	*n*	%	*n*	%	*n*	%
**Psychiatric diagnoses**						
Mental retardation†	2	1.7	1	1.1	1	3.5
ADHD	3	2.5	1	1.1	2	6.9
Psychotic disorders						
Substance-induced psychotic disorder	39	32.8	27	30.0	12	41.4
Schizophrenia	29	24.4	24	26.7	5	17.2
Other	6	5.0	4	4.4	2	6.9
Mood disorders						
Substance-induced mood disorder	19	16.0	14	15.6	5	17.2
Depressive disorders	16	13.4	15	16.7	1	3.5
Bipolar disorders	22	18.5	18	20.0	4	13.8
Other	1	0.8	1	1.1	0	0
Anxiety disorders						
Other than PTSD	6	5.0	6	6.8	0	0
PTSD	1	0.8	1	1.1	0	0
Cluster B personality						
Disorder	15	12.6	12	13.3	3	10.3
Traits	32	26.9	24	26.7	8	27.6
**Medical diagnoses**						
Head injury	27	22.7	19	21.1	8	27.6
Cardiovascular disease	18	15.1	14	15.6	4	13.8
HIV	7	5.9	6	6.7	1	3.5
Diabetes mellitus	5	4.2	4	4.4	1	3.5
Epilepsy						
Current	4	3.4	4	4.4	0	0
Previous	3	2.5	2	2.2	1	3.5

ADHD, Attention-deficit/hyperactivity disorder; HIV, Human immunodeficiency virus; PTSD, post-traumatic stress disorder.

† , Includes cases of low or borderline intelligence quotient or borderline intellectual functioning.

**TABLE 3:** Substances used (lifetime) in all patients accepted into the Substance Rehabilitation Unit during 2013–2014 (***n*** = 119).

Substances used	Accepted (*n* = 119)		Completers (*n* = 90)		Non-completers (*n* = 29)	
	*n*	%	*n*	%	*n*	%
**Downers (depressants)**	118	99.2	90	100	28	96.6
Cannabis	98	82.4	71	78.9	27	93.1
Alcohol	94	79.0	72	80.0	22	75.9
Illicit opioids (heroin, etc.)	18	15.1	12	13.3	6	20.7
Sedatives, hypnotics and anxiolytics	14	11.8	11	12.2	3	10.3
Methaqualone (mandrax, etc.)	9	7.6	5	5.6	4	13.8
Prescription opioids (opioid-containing analgesics, etc.)	7	5.9	6	6.7	1	3.5
Unspecified opioids	2	1.7	2	2.2	0	0
**Uppers (stimulants)**	48	4.0	33	36.7	15	51.7
Methcathinone (cat)	32	26.9	24	26.7	8	27.6
Cocaine (coke, crack, etc.)	28	23.5	19	21.1	9	31.0
Amphetamines and methamphetamines (tik, crystal, ecstasy, etc.)	24	20.2	18	20.0	6	20.7
**Other**						
Nicotine (cigarettes, snuff, etc.)	90	75.6	64	17.1	26	89.7
Nyaope and Whoonga	18	15.1	8	8.9	10	34.5
Other medication misuse (non-opioid-containing analgesics, antiretrovirals, etc.)	13	10.9	11	12.2	2	6.9
Hallucinogens (mushrooms, LSD, acid, Special K, etc.)	10	8.4	8	8.9	2	6.9
Inhalants and solvents (paint thinner, glue, etc.)	4	3.4	3	3.3	1	3.5
Other substance misuse	1	0.8	1	1.1	0	0

LSD, lysergic acid diethyamide.

Seventy-one per cent (*n* = 85) of the sample regularly used three or more substances. Eighty-two per cent (*n* = 79) of the 96 patients with a recorded age of debut used their first substance prior to the age of 18 years. Of the accepted patients with recorded prior rehabilitation attendance data (*n* = 115), 35% (*n* = 40) had attended some form of previous rehabilitation.

### Factors associated with completion status

Demographic factors found to be associated with completion of the SRU are summarised in Table 4. Disability grant status (*p* = 0.004) and level of education (*p* = 0.004) were the only two variables found statistically to be significantly associated with completion; patients with higher levels of education and those who had never received a disability grant were statistically more likely to complete rehabilitation (Table 4). However, only 32% (*n* = 38) of the cases had information on disability grant status; tests of association excluded cases without disability grant data.

**TABLE 4:** Demographic factors associated with completion of the Substance Rehabilitation Unit programme.

Demographic variables	*p*-value
Tertiary education (+) > matric (+) > Grade 8–11 (−) > Grade 1–7 (−)	0.004*
Disability grant status: Never (+) > current (−) > Previous (−)	0.004*
30–49 years (+) > 50 years (+) > under 30 years (−)	0.060
State patients (+) > voluntary patients (+) > assisted (−) > involuntary (−)	0.089
Female (+) > male (−)	0.141
Students and retired (+) > unemployed (+) > informal work (−) > employed (−)	0.359
Widowed (+) > divorced (+) > single (+) > other (=) > separated (−) > married (−)	0.534

(−), Negatively associated, that is, frequency of completion was lower than expected.

(+), Positively associated, that is, frequency of completion was higher than expected.

(=), Equivocal, that is, frequency of completion was as expected.

* , Statistically significant at the 0.01 level.

**FIGURE 1:**
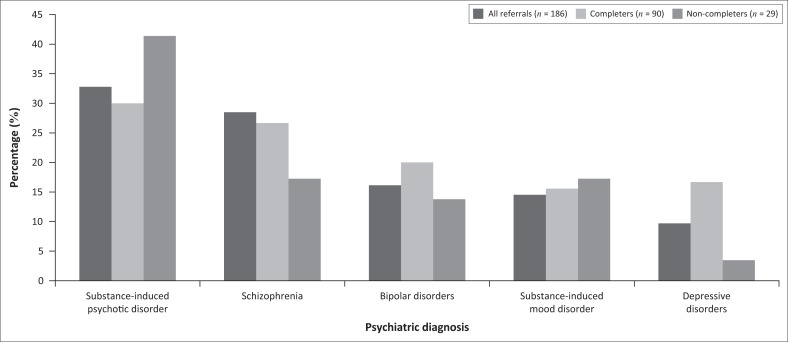
Differences between completers and non-completers in terms of the most common psychiatric diagnoses encountered in the sample.

No psychiatric or general medical conditions was found to be statistically significantly associated with completion of the SRU programme. In spite of the lack of statistical significance, differences between completers and non-completers can be noted for the most common psychiatric disorders (Figure 1). Non-completers had higher rates of substance-induced disorders, especially substance-induced psychotic disorder (41% vs. 30%), compared to completers. On the other hand, completers had higher rates of primary psychiatric disorders, such as schizophrenia (27% vs. 17%), bipolar disorders (20% vs. 14%) and depressive disorders (17% vs. 3%). Regarding general medical comorbidities, completers were less likely than non-completers to have had a previous head injury (21% vs. 28%), and more likely to have cardiovascular disease (16% vs. 14%) and be HIV positive (7% vs. 3.5%), but none of these were found to be statistically significant.

**FIGURE 2:**
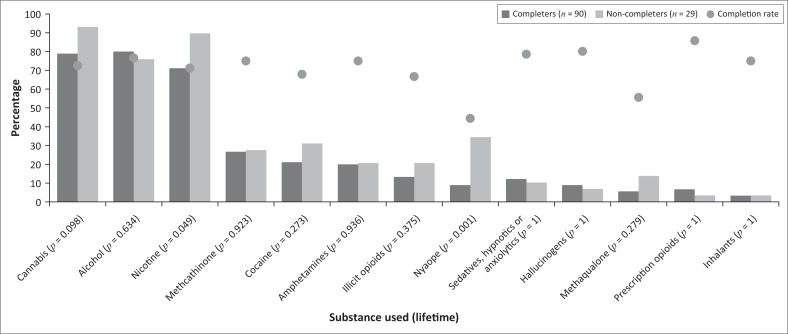
Frequency of substance use in completers and non-completers, and completion rates for each substance.

Figure 2 shows the frequencies of use for each substance in completers versus non-completers, as well as completion rates for each substance. Non-completers had higher frequencies of cannabis, illicit opioids, methaqualone, stimulants, nicotine, Nyaope and inhalant use; however, based on the statistics obtained, only Nyaope (***p*** = 0.001) and nicotine (***p*** = 0.049) use were significantly associated with non-completion. Although only 15% (*n* = 18) of the total sample had used Nyaope, non-completers had statistically significant higher rates of Nyaope use than completers (34.5% vs. 9%). Compared to other substances, Nyaope was associated with the lowest completion rate, with only 44% of users completing the programme. Nicotine use was also found to be statistically more frequent in non-completers than in completers (90% vs. 71%); however, 71% of users were still able to complete (Figure 2).

Likelihood of completion decreased when more substances were used (87.5% when only one substance was used vs. 73% when three or more substances were used); and increased with increasing age of debut (73% with an age of debut < 18 years vs. 80% with an age of debut > 25 years), but these were not found to be statistically significant. Non-completers were more likely to have had previous rehabilitation attempts as compared to completers, but this too was not found to be statistically significant (39% vs. 33%).

## Discussion

Completion rates were comparable to those in general substance rehabilitation centres locally and internationally,^17,30^ which is in line with international research showing that COD patients do not necessarily have higher rates of treatment non-completion than the general population.^6^

Studies show inconsistent associations of education with completion;^16^ the finding in this study that education was associated suggests that it may play a more important role in COD samples, where educational level often provides insight into the effect of mental illness on the level of functioning. The level of education was similar to that found in other South African studies, both in general and COD-specific substance rehabilitation centres.^7,17,24^ In contrast, unemployment was considerably greater than that found in general substance rehabilitation centres.^17,24^ However, similarly high levels of unemployment were found in other COD samples in South Africa.^7^ This finding may be related to our position within a public-sector psychiatric hospital.

From clinical experience, patients with serious mental illness in South Africa are sometimes denied disability grants when there is comorbid substance use, which may have a bearing on the low levels of disability grant receivers. However, this may also be explained by the fact that one-fifth of the sample (*n* = 21) were state patients who cannot receive grants because they are maintained by the state.^31^ As state patients also had the highest completion rates compared to other MHCA status groups, with 95% (*n* = 20) patients completing the programme, this may account for the association between not having a disability grant and completion (confounding bias). Further, there may be a non-response bias because of the amount of missing data for this variable. Non-completion of disability grant-holders may however also be related to another reason. Firstly, it may be related to lower functional status.^32^ Secondly, it may be related to secondary gain; disability grants are often the sole source of income for people with disabilities.^32^ In patients with mental illness who are reliant on social grants, the possibility of grants being cancelled upon symptom remission is a factor that has been linked to non-compliance.^33^ A third possibility is that grant non-receivers may be more motivated to complete the SRU programme in order to improve their chances of either finding employment or obtaining a disability grant,^34,35^ which suggests a likely incentive on the part of the patient to complete the SRU. Further research into the role of disability grants in substance rehabilitation in the COD population is indicated.

The lack of association between medical and psychiatric diagnosis, and completion is in keeping with existing research, with some exceptions.^6,16^ Firstly, this study did not find a significant association between intellectual disability and completion as shown in other studies.^16^ This may be explained by the small number of patients with intellectual disability who were accepted into the SRU (as it is a relative exclusion criteria for acceptance) and the assumption that those referred have already been deemed by their treating MDT to be intellectually suitable candidates for substance rehabilitation. Secondly, antisocial personality disorder and, to a lesser extent, histrionic personality disorder have been strongly associated with non-completion in international studies,^6,16^ but this was not the case in this study. The non-significant findings may be because we grouped cluster B disorders together and borderline personality disorder is not usually associated with treatment drop-out.^16^

Commonly used substances were similar to those found in other local COD samples,^5,7^ but prevalence rates far exceeded those in other studies.^17,24^ This is likely because of methodological differences, as we were unable to differentiate between primary and secondary substances, and we not only measured the substance for which patients were attending rehabilitation but also all the substances patients had used in their lifetime. In keeping with other studies, cannabis exceeded alcohol as the substance most often used amongst COD patients.^5,7,10,17^ It is worth noting that cannabis use has also overtaken alcohol use in non-COD samples in most South African regions.^24,27^ Perhaps, this is related to low cost, widespread availability, increasing community acceptance and assumption of safety of cannabis.

Although Nyaope was used by only 15% of the sample, it was significantly associated with non-completion; only 44% of the patients using it were able to complete the SRU programme. The rates of Nyaope use in our study are higher than in other local COD samples,^7,36^ which may be accounted for by differing geographical patterns or by increasing use with time. Nyaope is used by only 5% of patients in general substance rehabilitation centres.^24^ Further research into the association with non-completion is warranted.

The high prevalence of nicotine use is consistent with other studies. Patients with mental illness and those with SUD are more likely to use nicotine, use it more frequently and heavily, are less likely to quit and more likely to relapse into smoking than the general population.^37,38,39,40,41^ It has been referred to as the ‘hidden’ and ‘neglected’ epidemic amongst persons with mental illness.^40^ Nicotine may assist with cognition, attention, affect and sensory processing deficits in schizophrenia.^40,41^ Research has also shown that smokers with COD have poorer long-term outcomes, compared to non-smokers, which negatively affects both quantity and quality of life. Smokers have more psychiatric symptoms and substance use, more employment difficulties, more social problems, greater risk for suicide and higher mortality rates.^38,40,41,42^ Biological and psychosocial mechanisms likely play a role in this association.^3,4,5,40,41,43,44,45,46,47^ Smokers undergoing substance rehabilitation for other substances have higher rates of other substance use during rehabilitation, which may be associated with treatment failure, especially in cocaine use disorders.^43^ This may partly explain the finding in this study that nicotine use is negatively associated with completion.

## Limitations

Retrospective descriptive studies based on clinical records have inherent limitations, which are also evident in this study. As notes were not initially written for research purposes, records were at times unclear and difficult to translate onto the data capturing sheet. Certain variables would have been better obtained with other study methodologies, for example, by using structured interviews or standardised rating scales. Patterns of substance use, such as methods of use, severity of use and reasons for use, as well as psychosocial difficulties, severity of head injury and level of motivation could not be captured with the methodology of this study. Further, the use of standardised diagnostic instruments would have improved diagnostic certainty. Missing data, either because of untraceable files or poorly recorded variables, also impaired accurate data capturing and reduced sample sizes for certain variables. This limited the statistical analysis. This is particularly relevant to disability grant status, where results need to be interpreted with caution because missing data can result in a non-response or hidden bias. In smaller studies such as this one, either case or variable deletion is usually employed to deal with missing data.^48^ Although case deletion results in smaller sample sizes, we opted for this approach rather than variable deletion because the spread of missing data (which was minor in most cases) was distributed amongst most of the demographic variables.

There is also a notable selection bias as the study population was made up entirely of patients who had already gone through a referral and acceptance process based on predetermined inclusion and exclusion criteria to the SRU, as well as clinical judgement regarding motivation. These criteria were likely designed to take into account factors that may have an impact on completion. The findings can therefore not be generalised to patients who would not have met the criteria for inclusion into this SRU. The large number of state patients in the sample also limits generalisability.

Completion rates do not necessarily reflect similar levels of long-term abstinence. Completion rates of state and involuntary patients (under the MHCA) in particular may be misleading as patients may be motivated to attend substance rehabilitation for reasons other than lasting abstinence, such as a change in their usual routines, or the hope that they will be discharged sooner or receive other benefits (secondary gain). Some patients may complete the programme because they assume it is expected of them as part of their otherwise involuntary treatment. Despite thorough assessment of motivation prior to acceptance into the SRU, this possibility remains and limits interpretation of the non-significance of MHCA status on completion status. Furthermore, MHCA status was captured only once according to documented MHCA status on admission; the study did not assess whether patients were formally reassessed for MHCA status at any point during admission or prior to referral to the SRU and therefore may not accurately reflect MHCA status at the time of referral to the SRU. In hospitals where patients are routinely reassessed for MHCA status at the time of referral to a substance rehabilitation programme, it may result in different rates of involuntary patients being referred. Generalisability may therefore be limited. Nevertheless, it is encouraging that admission MHCA status was not found to be significantly associated with completion status.

Another limitation is the focus of this study on individual patient and substance risk factors. The interplay between patient factors and service factors, such as perception of treatment, patient–therapist match and therapeutic alliance, may be more important than individual patient factors taken in isolation; patient factors alone may play more of a moderating role.^16,30,49^

## Strengths and contributions

Firstly, there are few substance rehabilitation programmes in South Africa that cater specifically for patients with COD, and therefore studies on the outcomes and effectiveness of such programmes are valuable.^15^ This is the first study in South Africa (to the authors’ knowledge) to examine completion rates and factors associated with completion of a substance rehabilitation programme in psychiatric inpatients.

Secondly, this is the first study to our knowledge examining the role of Nyaope in substance rehabilitation completion in COD samples, and only a few other studies have looked at the role of disability grants in mental illness, substance use or in substance rehabilitation. The role of nicotine use in substance rehabilitation in South Africa is also not well researched yet.

Thirdly, a control group of non-completers allowed for more meaningful analysis.

This study therefore adds to the overall understanding of substance rehabilitation in COD samples in South Africa and allows for some recommendations to be made.

## Recommendations

Psychiatric facilities that are endeavouring to incorporate substance rehabilitation services for patients with COD should consider the following:

- Further research is needed to determine whether voluntary substance rehabilitation improves long-term abstinence rates in COD inpatients that are classified as involuntary or state patients under the MHCA.
- Patients with lower education or on a disability grant, as well as Nyaope and nicotine users, may be at higher risk for non-completion; however, further research is needed to confirm these findings in other settings. Increased support and a greater emphasis on treatment alliance, motivational interviewing, problem-solving, and coping skills may be helpful to improve treatment outcomes in patients with risk factors for non-completion,^14,16,50^ but further research is recommended to identify effective strategies, especially in light of resource limitations.
- Further research should focus on the role of disability grants in substance rehabilitation for patients with COD.
- We concur with international guidelines that emphasise the need for health care workers to assess for smoking in patients with COD and follow evidence-based treatments to assist cessation.^41^ The American Psychiatric Association suggests that the period of psychiatric admission is an ideal time to quit smoking, and offers practical guidelines in this regard.^37,38,40,51,52^

## Conclusions

Substance rehabilitation for patients with COD is challenging. Outcomes may be improved, and the inefficient use of limited treatment resources avoided, by identifying patients who are at risk of non-completion so that targeted interventions may be applied. This study has yielded several results in areas that have not yet been well researched in South Africa. Risk factors for non-completion may include lower levels of education, being on a disability grant and using Nyaope or nicotine, but may vary in different settings. Future research should focus on identifying further factors that may affect completion of substance rehabilitation in local COD patients, the role of disability grants in COD patients, and the effect of Nyaope and nicotine use on COD substance rehabilitation. Effective and accessible interventions to assist vulnerable patients also need to be identified.
